# Life of double minutes: generation, maintenance, and elimination

**DOI:** 10.1007/s00412-022-00773-4

**Published:** 2022-04-30

**Authors:** Mila Ilić, Irene C. Zaalberg, Jonne A. Raaijmakers, René H. Medema

**Affiliations:** 1 Division of Cell Biology, Oncode Institute, The Netherlands Cancer Institute, Plesmanlaan 121, 1066 CX Amsterdam, The Netherlands; 2 Center for Molecular Medicine, University Medical Center Utrecht, Utrecht University, Universiteitsweg, 100, 3584 CG Utrecht, The Netherlands

**Keywords:** Double minutes, ecDNA, Extrachromosomal DNA, Extrachromosomal oncogene amplification, Gene amplification

## Abstract

Advances in genome sequencing have revealed a type of extrachromosomal DNA, historically named double minutes (also referred to as ecDNA), to be common in a wide range of cancer types, but not in healthy tissues. These cancer-associated circular DNA molecules contain one or a few genes that are amplified when double minutes accumulate. Double minutes harbor oncogenes or drug resistance genes that contribute to tumor aggressiveness through copy number amplification in combination with favorable epigenetic properties. Unequal distribution of double minutes over daughter cells contributes to intratumoral heterogeneity, thereby increasing tumor adaptability. In this review, we discuss various models delineating the mechanism of generation of double minutes. Furthermore, we highlight how double minutes are maintained, how they evolve, and discuss possible mechanisms driving their elimination.

**Supplementary Information:**

The online version contains supplementary material available at 10.1007/s00412-022-00773-4.

## Introduction

**Fig. 1:**
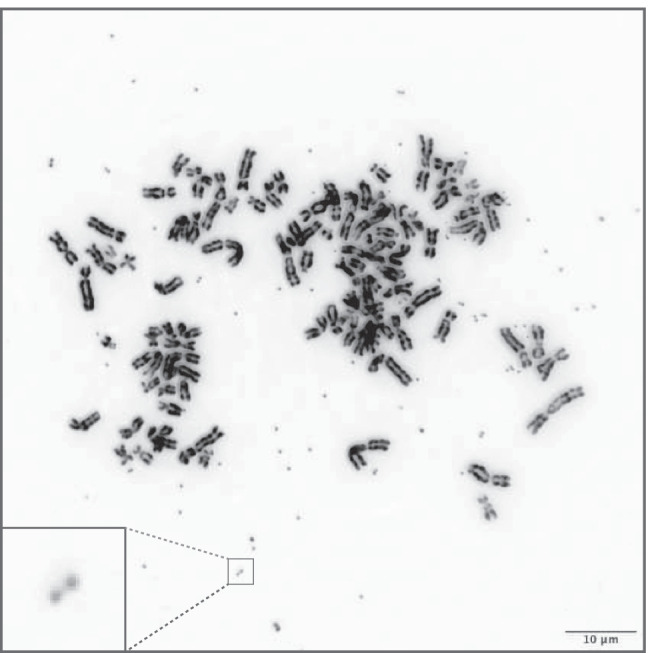
Double minutes in HeLa cell line. Double minutes are derived through inducing drug resistance

Extrachromosomal DNA molecules known as double minute chromosomes are commonly detected in cancer but not in healthy tissue (Turner et al. 2017; Kim et al. 2020). Double minutes are gene-containing, circular DNA molecules (Hamkalo et al. 1985; Maurer et al. 1987; Wu et al. 2019), that are relatively large structures, typically ranging from ~ 100 kilobases up to several megabases in size (Wu et al. 2019; Koche et al. 2020). Of note, double minutes are also referred to as ecDNA or cancer-associated ecDNA and differ from extrachromosomal circular DNA (eccDNA) structures such as telomeric circles, small polydispersed DNA elements, and microDNAs. EccDNA molecules are smaller than double minutes (usually less than 1 kb), do not contain full genes, and are found in both healthy and tumor cells (Verhaak et al. 2019; Koche et al. 2020). Further, in contrast to cancer-associated neochromosomes, giant supernumerary chromosomes that occur in circular as well as linear form that contain functional centromeres and possibly functional telomeres (Garsed et al. 2014), double minutes lack these typical chromosomal elements (Levan et al. 1976; Levan and Levan 1978; Lin et al. 1990). The term double minutes stems from the first cytogenetic analysis reports (Spriggs et al. 1962) where small/minute chromatin bodies were observed in metaphase spreads of cancer cells often found as paired/double structures, as exemplified in Fig. 1. Later, these extrachromosomal gene amplifications were frequently reported to exist in both treated and untreated tumors (Röijer et al. 2002; Gibaud et al. 2010; Rausch et al. 2012; Nones et al. 2014; L′Abbate et al. 2018; deCarvalho et al. 2018; Xu et al. 2019; Kim et al. 2020; Zhao et al. 2021), and it was shown that cancer cells can contain up to hundreds of such extrachromosomal DNA molecules (Turner et al. 2017), which frequently carry well-known oncogenes, e.g., *MDM2*, *MYC*, and *EGFR* (Kim et al. 2020).

Although the first description of double minutes/ecDNA dates back to the 1960s, their widespread occurrence in cancer was only recently recognized. The rise of genome sequencing at the end of the twentieth century led to the identification of common gene amplifications in cancer genomes. However, insufficient sequencing depth, bulk sequencing, and the assumption that detected amplifications were intrachromosomal made that double minutes went largely unnoticed (Mullard 2020) and were commonly misassigned as being focal amplifications (Zack et al. 2013; Krijgsman et al. 2014). Various optimizations facilitated their detection—computational breakthroughs allowed inferring circularity from short-read sequences (Turner et al. 2017; Deshpande et al. 2019), while innovative methods allowed for physical separation of double minutes (Koche et al. 2020; Hung et al. 2021a). These detection methods were augmented by optical mapping and long-range sequencing (Wu et al. 2019; Luebeck et al. 2020). Employing optimized sequencing data analysis methods, double minutes were detected in nearly half of all tumor cell lines tested (Turner et al. 2017). Analysis of over 5000 human tumor samples from The Cancer Genome Atlas (TCGA) and the Pan-Cancer Analysis of Whole Genomes (PCAWG) confirmed that the high frequency of double minutes detected in tumor cell lines was also of relevance in patients, as they were detected in 25 out of 29 analyzed tumor types (Kim et al. 2020). Overall, ~ 14% of analyzed samples contained double minutes/ecDNA, with the highest incidence reported in glioblastoma (~ 60%), sarcoma (~ 48%), and esophageal carcinoma (~ 37%) (Kim et al. 2020).

Extrachromosomal gene amplification can provide significant benefits to cancer cells in comparison to intrachromosomal amplifications. First, oncogenes amplified extrachromosomally, i.e., on double minutes, can reach higher copy numbers, with up to a hundred gene copies per cell (Turner et al. 2017). Second, the co-amplification of enhancers, sometimes from different topologically associated domains, as well as an accessible chromatin structure and possible *trans* interactions among double minutes may result in increased gene expression (Morton et al. 2019; Wu et al. 2019; Kim et al. 2020; Zhu et al. 2021). Together, the high copy number and transcription-promoting properties of double minutes result in increased levels of cancer-promoting proteins (Trent et al. 1986; Storlazzi et al. 2006; Koche et al. 2020; Yi et al. 2021). Third, the absence of centromeres on double minutes and their consequent unequal distribution over daughter cells upon cell division increase tumor evolution and adaptability, thereby rendering tumors better equipped to cope with changes in the environment and to rapidly acquire resistance to anti-cancer therapies (Nathanson et al. 2014; Turner et al. 2017; deCarvalho et al. 2018). Together, these properties of double minutes lead to decreased survival probability for patients carrying gene amplifications on extrachromosomal DNA compared to patients with intrachromosomal or no amplifications (Kim et al. 2020).

All in all, the widespread implications of double minutes/ecDNA in cancer are becoming increasingly clear. However, insights into the mechanisms underlying fundamental processes of double minute biology are limited. Here, we provide a comprehensive review of both former and recent literature describing how double minutes are formed, maintained, and eliminated in cancer cells, as well as discuss future research directions.

## Generation of double minutes

Multiple mechanisms have been proposed to explain the generation of double minutes. Here, we broadly divide the proposed mechanisms into (1) “simple” formation that leaves chromosomes largely intact and (2) their formation as a consequence of chromothripsis, which is considered “complex” chromosome restructuring and is accompanied by gross chromosomal rearrangements.

### Generation of double minutes with no or limited chromosomal rearrangements

Numerous studies reported the presence of double minutes in cells containing no apparent or very limited chromosomal scars, which suggests the existence of a “simple” form of double minute generation that does not involve chromosomal catastrophe. In some instances, this “simple” double minute generation was accompanied by preservation of the corresponding chromosomal sequence (Toledo et al. 1993; Vogt et al. 2004, 2014; Storlazzi et al. 2006, 2010; L’Abbate et al. 2014), but in most cases, it was described to be paired with deletion of the corresponding sequence from its original chromosomal location (Carroll et al. 1988; Ruiz and Wahl 1990; Toledo et al. 1993; Coquelle et al. 1997; Röijer et al. 2002; Storlazzi et al. 2006, 2010; deCarvalho et al. 2018). Here, we refer to these subtypes of “simple” double minute generation as non-deletion-associated double minute generation and deletion-associated double minute generation, respectively.

**Fig. 2:**
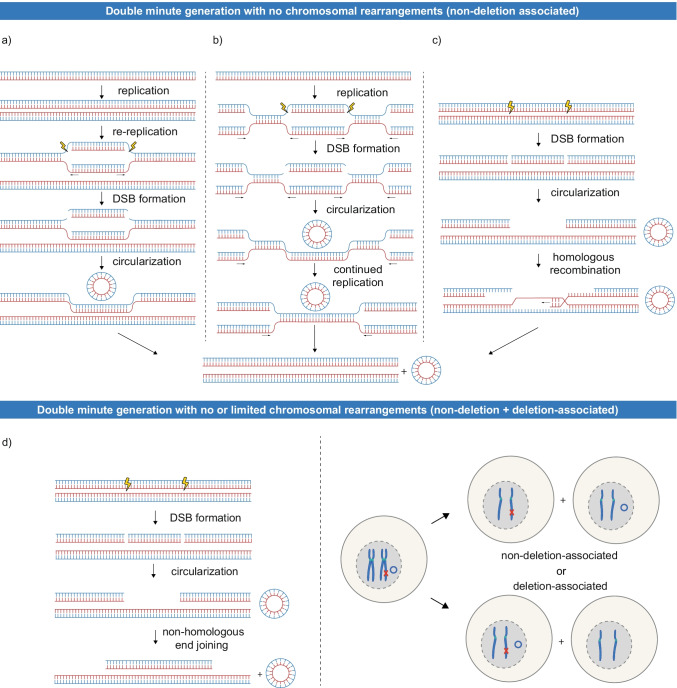
Proposed models of “simple” double minute generation with no or limited chromosomal rearrangements. Top: Three models were proposed to explain non-deletion-associated double minute generation: **a** Re-replication caused by origin refiring is followed by excision of DNA fragments and their circularization (replication – re-replication – excision). **b** In a model summarized as replication – excision – continued replication, stalling and destabilization of the replication forks would lead to excision of a DNA fragment. Repair of the stalled replication forks could happen through breakage-induced repair mechanisms. For simplicity, we depict the continued replication simply as further advancement of replication forks. **c** DNA damage on one of the sister chromatids leads to excision of a DNA fragment, followed by repair through homologous recombination (post-replicative excision – homologous recombination). Excision has been depicted here as two DSBs. Bottom: **d** A model explaining the non-deletion- and deletion-associated generation by a single mechanism. Double minutes are generated in G_2_ phase by excision of a DNA fragment followed by circularization through non-homologous end joining. Upon cell division, the double minute can end up in the same daughter cell as the intact chromatid (non-deletion-associated generation) or as the chromatid with the deletion (deletion-associated generation). In case of the non-deletion-associated phenotype, negative selection of the cell harbouring the deletion can lead to it not being detected

Several models have been proposed to explain the mechanisms behind the non-deletion-associated and deletion-associated generation of double minutes. Very early on, DNA overreplication was proposed to account for the generation of non-deletion-associated double minutes (Mariani and Schimke 1984; Hill and Schimke 1985). Replication followed by unscheduled origin refiring in G_2_/M (Mazurczyk and Rybaczek 2015) and subsequent recombination or DNA breakage could give rise to double minutes consisting of the re-replicated DNA (*replication – re-replication – excision*, *Fig. *2a). Re-replication-induced gene amplification (RRIGA) was experimentally confirmed, although generation of double minutes through this mechanism remains elusive (Green et al. 2010). Another model, proposing stalled replication forks as intermediates of double minute generation (Wahl 1989; Vogt et al. 2004), we summarize in a simplified scheme as *replication – excision – continued replication* (Fig. 2b). This replication-coupled mechanism could originate in under-replicated regions, where stalled and destabilized replication forks would lead to double-strand break (DSB) formation and excision of a DNA fragment. Studies reporting how duplications arise as a consequence of stalled replication, implicating breakage-induced repair mechanisms (BIR, MiDAS), are put forward (Costantino et al. 2014; Macheret et al. 2020), and we imagine they could be extended to explain the formation of double minutes. Lastly, a model where DNA is excised in the G_2_ phase of the cell cycle was postulated. In contrast to the previous models, the post*-*replicative excision model can account for both non-deletion-associated and deletion-associated generation. In this model, excision of a chromosomal fragment after replication leaves one sister chromatid unaffected, while creating a deletion on the other sister. If the formed double minute is segregated to the daughter cell that receives the intact sister chromatid, the non-deletion-associated phenotype is established. Conversely, in case the formed double minute ends up in the same cell as the affected sister chromatid, this gives rise to the deletion-associated phenotype (Fig. 2d) (Roelofs et al. 1992; Vogt et al. 2004). Alternatively, DNA repair through homologous recombination after G_2_ excision could also result in the non-deletion-associated generation of double minutes (Fig. 2c).

So far, these models are largely speculative, and limited experimental data exists to support them. It is still unclear to what extent each model contributes to the generation of double minutes or whether other “simple” forms of double minute generation exist. Depending on the model system used, both non-deletion- and deletion-associated double minute generation have been observed to be exclusively present, whereas in other studies, both types were detected side-by-side (Table 1). As the overview of reported phenotypes shows, various mechanisms of “simple” double minute generation may exist, and cellular context could influence which mechanism(s) is actualized.

**Table 1:**
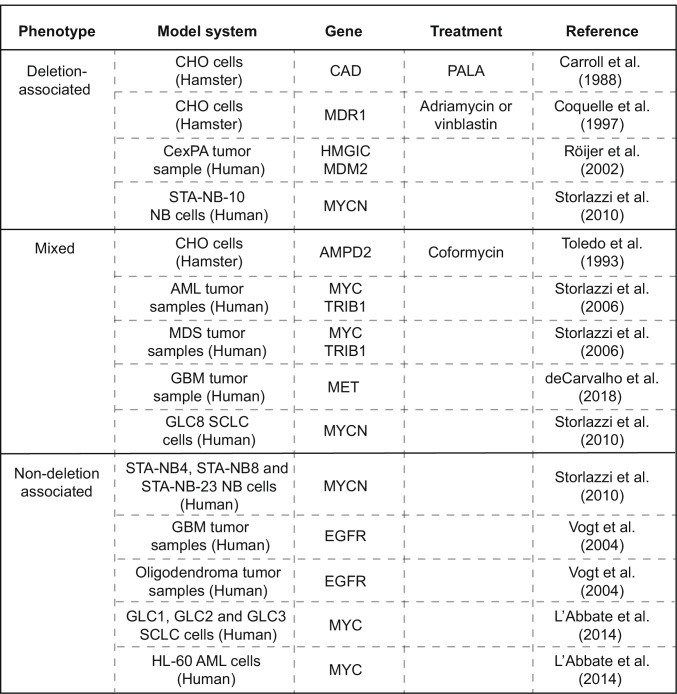
Overview of reported phenotypes in studies describing “simple” generation of double minutes *CexPA* carcinoma ex pleomorphic adenoma, *NB* neuroblastoma, *AML* acute myeloid leukemia, *MDS* myelodysplastic syndrome, *GBM* glioblastoma, *SCLC* small cell lung cancer

A commonality of all the models is that the excision of circular fragments is initiated by DSBs. A large number of chemical and physical agents are known to induce DNA damage, including clastogenic drugs or γ-irradiation, two frequently used anti-cancer therapies. For instance, double minute generation was observed in cells treated with chemotherapeutics such as actinomycin D and adriamycin (Coquelle et al. 1997, 1998). In addition, cancer-intrinsic properties, such as hypoxia and replication stress, can also lead to DNA breakage (Zeman and Cimprich 2014). Although early studies suggested that breakpoints resulting in the generation of double minutes frequently localize to specific genomic regions, such as CpG islands (Rizwana and Hahn 1998; Foureman et al. 1998) or fragile sites (genomic loci that are prone to DSBs) (Coquelle et al. 1998), a recent study investigating double minutes harboring frequently amplified oncogenes revealed breakpoints to occur at random around the oncogene (Kim et al. 2020). However, this study mainly included non-treated tumors, and it cannot be excluded that certain genomic regions are more prone to double minute generation upon exposure to specific DNA damaging agents.

### Generation of double minutes through chromothripsis

Besides “simple” generation, double minutes can form through chromothripsis, a catastrophic event in which one or more chromosomes are shattered into numerous DNA fragments (Stephens et al. 2011). The DNA fragments formed in this process can be “stitched” back together in a random order to form a derivative of the shattered chromosome(s) (Stephens et al. 2011). Instead of incorporating into a chromosomal derivative, DNA fragments can also self-ligate or ligate to a few other DNA fragments to form double minutes, a phenomenon that has been repeatedly reported since the discovery of chromothripsis in 2011 (Gibaud et al. 2010; Stephens et al. 2011; Rausch et al. 2012; Nones et al. 2014; Kim et al. 2020; Rosswog et al. 2021; Shoshani et al. 2021). Of note, most studies describing the deletion-associated “simple” double minute generation exclusively analyzed chromosome structure based on cytogenetic observations such as chromosome-banding patterns and fluorescence in situ hybridization (FISH). Therefore, it is easy to imagine that the presence of chromosomal abnormalities was overlooked in some of these studies and that chromothripsis drove double minute generation (Ly and Cleveland 2017).

**Fig. 3:**
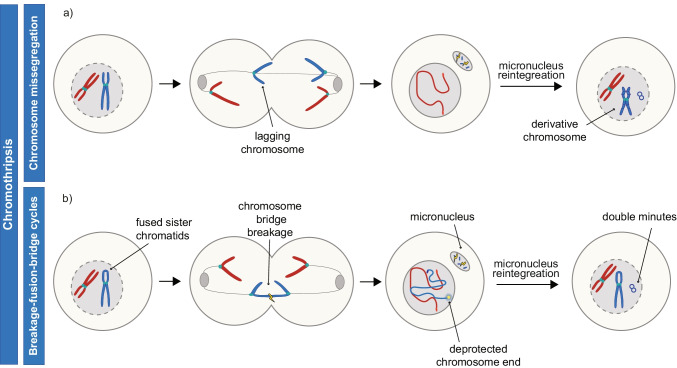
Mechanisms of double minute generation through chromothripsis. **a** Chromothripsis is caused by lagging chromosome micronucleation. Often, a derivative chromosome is formed in this process. **b** Breakage-fusion-bridge cycles can result in generation of double minutes when a chromosome bridge is shattered. Here, there are two possible scenarios. A micronucleus-independent process, where the damage occurring at the site of the bridge breakage leads to double minute generation. Alternatively, micronucleation could precede double minute formation

There are two major routes leading to chromothripsis: (1) micronucleation of missegregated chromosomes and (2) chromatin bridge formation (reviewed in Ly and Cleveland 2017; Marcozzi et al. 2018). When a chromosome lags during anaphase, it can be excluded from both daughter cell nuclei and end up in a micronucleus instead (Crasta et al. 2012; Ly et al. 2019). Due to micronuclear membrane rupture, DNA damage occurs and causes chromothriptic shattering of the chromosome. If the micronucleus contents are taken up by the primary nucleus in the subsequent cell division, the fragments will be randomly ligated, a process often accompanied by circularization of individual or multiple fragments (Fig. 3a) (Zhang et al. 2015). Thus, micronuclear shattering of a missegregated chromosome provides a mechanism of double minute generation through chromothripsis.

Another type of mitotic error, chromosome bridges, can be formed as a result of unresolved DNA catenations, replication or repair intermediates, or dicentric chromosomes. Here, we focus on the role of dicentrics that lead to breakage-fusion-bridge (BFB) cycles, since most of the reports connecting double minute formation involving bridge formation were described in the context of BFB cycles. In BFB cycles, telomere loss or telomere deprotection can lead to fusion of replicated sister chromatids at the chromosome end (McClintock 1941; Lo et al. 2002; Stroik and Hendrickson 2020). In the following cell division, the fused sister chromatids cannot separate which produces a DNA bridge in anaphase, that is eventually torn apart by mechanical forces (Janssen et al. 2011; Umbreit et al. 2020) or nuclease activity (Maciejowski et al. 2015). This results in a DSB near the ends of previously fused chromatids, which once again deprotects the chromosome ends and initiates a new BFB cycle (McClintock 1938, 1941). This process continues until a chromatid captures a telomere or is shattered through chromothripsis. Of note, recently important revisions were made to the BFB model, additionally underscoring the interconnection of BFB cycles and chromothripsis (Umbreit et al. 2020). Further, it was reported that the breaks in BFB cycles do not always occur in the telomere vicinity, but can also take place more distally in chromosome arms (Umbreit et al. 2020; Hintzen et al. 2021). Double minutes can form as a consequence of BFB cycles, either through (self-)ligation of broken DNA fragments generated at the site of bridge breakage or upon fragment micronucleation followed by re-incorporation into the primary nucleus (Fig. 3b) (Toledo et al. 1992; Singer et al. 2000; Rausch et al. 2012; Nones et al. 2014; Shoshani et al. 2021). Accumulating evidence suggests that it is the BFB-mediated breakdown of regions containing intrachromosomal amplification that leads to double minute generation (discussed in section “The complex relationship of double minutes and HSRs”).

### Circularization of generated DNA fragments 

Independent of which model(s) are correct and what the extent of their contribution to double minute generation is, the excision of extrachromosomal DNA fragments is coupled with or followed by their circularization. To establish circularity, the excised DNA fragments must be (self-)ligated. This circularization could be mediated by two main DSB repair mechanisms: (1) homologous recombination (HR) and (2) non-homologous end joining (NHEJ) (Chapman et al. 2012). Of these two, HR is a repair pathway dependent on the presence of sequence homology, used in S and G_2_ phases to ensure error-free DNA repair (Rothkamm et al. 2003; Chapman et al. 2012). In contrast, NHEJ mediates repair by direct ligation of DSB ends, which is more error-prone and can result in deletions or small insertions at the ligation junction, although an error-free outcome is not excluded (Chang et al. 2017).

Completion of DNA excision could happen through recombination when a single DSB would be repaired using a homologous sequence, therefore instantly yielding a circular DNA molecule. As the junction sequence formed upon ligation is absent from chromosomal DNA, HR would only be possible if there were homologous sequences at the ends of the DNA fragments themselves. However, the absence of homologous regions surrounding junctions within double minutes argues against such a mechanism (Vogt et al. 2004; Storlazzi et al. 2006, 2010; Gibaud et al. 2010; Rausch et al. 2012; L’Abbate et al. 2014; Kim et al. 2020). Therefore, the generation of double minutes is likely initiated by the excision of linear DNA fragments, which are subsequently circularized through ligation of the open ends. For both “simple” and chromothripsis-associated generation of double minutes, NHEJ is the probable DNA repair pathway employed in the circularization of excised linear DNA fragments (Vogt et al. 2004; Storlazzi et al. 2006; Gibaud et al. 2010; Rausch et al. 2012; Zhu et al. 2013; L’Abbate et al. 2014; Kim et al. 2020; Shoshani et al. 2021). Indeed, inhibition of NHEJ decreased the frequency of double minute generation (Shoshani et al. 2021). Moreover, microhomologies and short, non-templated insertions, which are typical by-products of NHEJ, are frequently present at double minute junctions (Vogt et al. 2004; Gibaud et al. 2010; Rausch et al. 2012; Zhu et al. 2013; L’Abbate et al. 2014; Shoshani et al. 2021). This frequent detection of microhomologies suggests that, next to canonical NHEJ, microhomology-mediated end joining (MMEJ) also plays a role in circularization (Seol et al. 2017).

## Maintenance of double minutes

### Double minute replication and separation of sisters

Double minutes are maintained over generations of cells by a mechanism of replication that resembles that of chromosomal DNA. Mirroring the experiments of Meselson and Stahl on chromosomal DNA (Meselson and Stahl 1958), double minute-DNA from cells grown in BrdU for one generation was of hybrid buoyant density, suggesting that BrdU was incorporated only in one DNA strand and double minutes had replicated once. After the second generation in BrdU, half of the double minutes were of hybrid and the other half of high density, again consistent with one round of replication (Carroll et al. 1987; Von Hoff et al. 1988; Ruiz et al. 1989). Moreover, as seen for chromosomal DNA (“Harlequin chromosomes”), differential staining was observed between the paired DNA structures within a double minute after two generations of BrdU-labeling (Barker et al. 1980; Takayama and Uwaike 1988). Replication of double minutes was found to take place in early to mid-S phase (Lubs et al. 1966; Levan et al. 1978; Barker et al. 1980; Mariani and Schimke 1984; Itoh and Shimizu 1998) and to be associated with the relocation of double minutes from the nuclear periphery to the inner regions of the nucleus (Itoh and Shimizu 1998). One could speculate that the high intratumoral heterogeneity in double minute numbers is in part caused by unscheduled replication independent of the chromosomal DNA, in addition to their uneven segregation in mitosis (see the next section). However, the evidence presented so far suggests that double minutes replicate similarly to chromosomal DNA: once per cell cycle and in S phase.

Based on premature chromatin condensation experiments (PCC, where cells in different cell cycle stages are fused with mitotic cells to induce chromatin condensation), it was concluded that double minutes are maintained as pairs post-replication, in S and G_2_ cell cycle phases (Takayama and Uwaike 1988). Due to the absence of centromeres, the two copies of DNA that make a double minute are not segregated by default during mitosis. Although it is widely considered that the majority of the double minute sisters have separated by the end of mitosis, such quantifications are based on metaphase spreads, and it has long been reported that colchicine treatment (used to arrest cells in metaphase when making spreads) stimulates separation of sisters double minutes (Levan et al. 1978; Kanda et al. 1998). These quantifications could be additionally misled by chromatin connections between sisters being broken when dropping the cells onto the slides (Takayama and Uwaike 1988; Jack et al. 1987). In absence of drugs inducing mitotic arrest, double minutes (near-) exclusively appear as pairs during mitosis (Jack et al. 1987; Kanda et al. 1998, 2001). A significant fraction of double minutes may remain paired even after mitosis, as paired structures were observed in the early G_1_ phase in live imaging experiments of lacO-tagged double minutes (Kanda et al. 2001). Similarly, paired double minutes with differential BrdU-labelling pattern — indicative of non-disjunction — were detected in G_1_ phase cells in PCC experiments, although this pairing appeared restricted to early G_1_ (Barker et al. 1980; Takayama and Uwaike 1988).

Replicated chromosomal DNA is held together by a ring-shaped protein complex called cohesin. Cohesin is displaced from chromosome arms in prophase, but it is retained on centromeres until the metaphase-to-anaphase transition (Haarhuis et al. 2014). Since double minutes do not contain centromeres, most cohesin is likely removed in prometaphase. In addition to cohesin, DNA intertwinings between sister chromatids, named catenanes, keep the sisters together (Farcas et al. 2011). All catenanes connecting the chromosomal sister chromatids are resolved in late mitosis by topoisomerase IIα when tension is created (Farcas et al., 2011). As double minutes are not segregated by spindle pulling forces, catenanes may remain. This resembles the reports in fruit fly that show acentric sisters to remain associated well after separation of the intact chromosomes, through DNA catenations (Vicars et al. 2021). Indeed, chromatin fibers connecting double minutes were reported (Jack et al. 1987; Deng et al. 2006). However, the exact nature and molecular composition of these fibers need further investigation. Altogether, a likely explanation for the continued linking of double minutes in mitosis is the presence of residual catenanes. Alternative explanations, such as remnant cohesin molecules, involvement of proteins associated with ecDNA hubs (see the next section), and the action of yet unknown double minute-binding proteins, can also not be excluded.

**Fig. 4:**
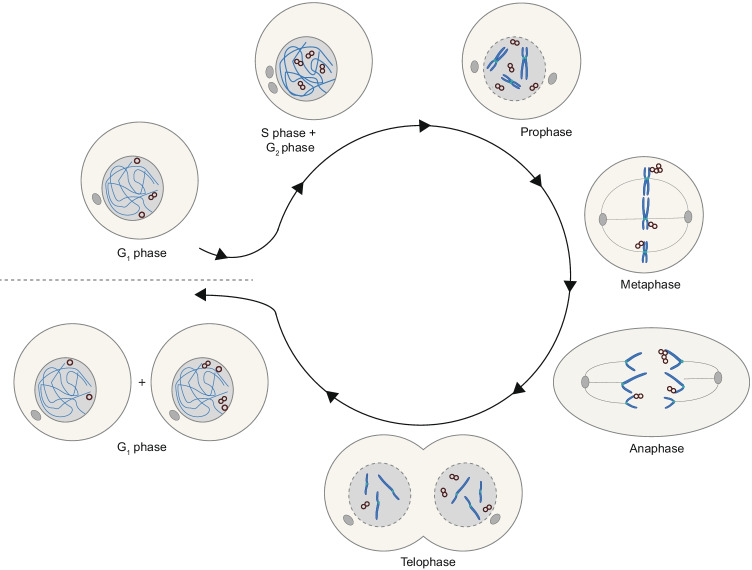
Proposed model for the behaviour of double minutes throughout the cell cycle. Double minutes are replicated during S phase to form paired structures. In mitosis, double minutes tether to chromosomes at metaphase and are found in proximity of the chromosome tips in anaphase. Sister double minutes remain paired during G_2_ and mitosis, and their mitotic nondisjunction results in unequal distribution of double minutes over daughter cells

Here, we summarize a model wherein double minutes are normally replicated in S phase and the majority of double minute copies remain paired until early G_1_ phase (Fig. 4).

### Segregation of double minutes in mitosis and their gene expression

Although double minutes are replicated once per cell cycle, their mitotic non-disjunction and asymmetric segregation can drive fast accumulation of up to tens to hundreds of double minutes per cell (Turner et al. 2017). In prometaphase, double minutes form clusters that localize to the periphery of the chromosome rosette (Levan and Levan 1978; Shimizu et al. 1998; Kanda et al. 2001). These clusters remain at the chromosomal periphery in metaphase and are found to associate with chromatid tips during anaphase while chromosomes segregate, a phenomenon known as “hitchhiking” (Levan and Levan 1978; Hamkalo et al. 1985; Tanaka and Shimizu 2000; Kanda et al. 2001; Shimizu et al. 2007). Finally, double minutes distribute unevenly and randomly to daughter cells (Fig. 4) (Kanda et al. 1998; Turner et al. 2017; Lange et al. 2021; Yi et al. 2021). Likely, mitotic tethering contributes to the uneven distribution of double minutes over daughter cells upon cell division. This could happen already as a consequence of chromatid-double minute associations being distributed unevenly over chromatids that end up in different daughter cells. In addition, if a cluster of double minutes is attached to both sister chromatids, it can be pulled in two directions which results in the formation of DNA bridges consisting of “strings” of double minutes (Kanda et al., 1998). With the progression of mitosis, these bridges are eventually broken. If the breakage is asymmetrical, double minutes are distributed unevenly over daughter cells, thereby further advancing cell-to-cell heterogeneity in copy number (Levan and Levan 1978; Kanda et al. 1998, 2001; Tanaka and Shimizu 2000).

The exact mechanisms by which double minutes tether to host chromatids remain largely unknown. Of interest, tethering to host cell chromosomes was also observed for genomes of several double-stranded DNA viruses including papillomavirus, Epstein-Barr virus, and Kaposi’s sarcoma-associated herpesvirus (Coursey and Mcbride 2019). Here, the tethering is mediated by interactions of viral proteins and various host cell proteins, suggesting that the tethering of double minutes to chromosomes also requires action of specific proteins. On the other hand, fibers connecting double minutes to chromosomal DNA were observed even in samples removed of proteins (Deng et al. 2006).

Noteworthy, a role for the bromodomain and extraterminal domain (BET) protein family member Brd4 was repeatedly identified in tethering of various viral genomes (You et al. 2004, 2006; Lin et al. 2008). Recently, Brd4 was shown to co-localize with clusters of *MYC*-encoding double minutes observed in interphase. Moreover, these double minute clusters dispersed upon treatment with the BET-inhibitor JQ1 (Hung et al. 2021b). It is tempting to speculate that, similar to viral genomes, double minutes depend on Brd4 for attachment to mitotic chromosomes. Whether Brd4 is indeed a factor responsible for tethering double minutes to chromosomes, and/or which other proteins fulfill this function, awaits further research. It is possible that the specific players enabling hitchhiking vary per cell line, cancer-type, or are double minute-type-specific, as described for viral episomes.

**Fig. 5:**
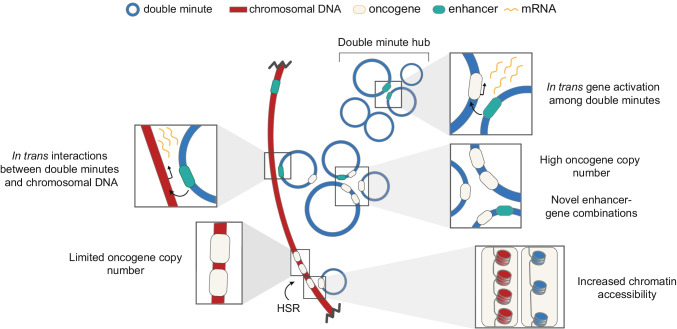
Unique modes of double minute-gene expression. Double minutes reach higher gene copy number when compared to intrachromosomal gene amplification in homogeneously staining regions (HSRs). Even when normalized to copy number, transcriptional output of double minutes could be higher as (1) double minute chromatin is more accessible; (2) double minute formation may lead to novel *in cis* regulation of oncogenes through incorporation of enhancer-gene pairs that normally localize to different topologically associated domains; (3) genes on double minutes may be activated *in trans* by enhancers on different double minutes within transcriptional hubs. Interestingly, gene activation *in trans* may also result in global increased expression of chromosomal genes

As mentioned, double minute clustering was also described in interphase, and here, they significantly correlated with transcription probability, suggesting the clusters act as transcription hubs (Hung et al. 2021b). Indeed, hubs of double minutes colocalize with hyperphosphorylated RNA polymerase II, indicating they are likely sites of active transcription (Yi et al. 2021). Possibly, the increased transcription in hubs is caused by intermolecular interactions between various double minutes, allowing enhancers to activate gene transcription in *trans* (Zhu et al. 2021; Hung et al. 2021b). Thus, apart from the intrinsic properties of double minutes such as higher copy number and increased chromatin accessibility compared to chromosomal DNA, intermolecular association of double minutes is proposed to provide an additional way of enhancing expression of genes on double minutes but also to affect global gene expression through chromosomal-double minute interactions (Zhu et al. 2021). In Fig. 5, we summarize the unique properties proposed to enable higher transcriptional output of double minutes in comparison to intrachromosomal amplification, even when normalized per copy number (recently reviewed in Wu et al. 2022). So far, the universality of these mechanisms remains an open question — they could prove to be cell line and perhaps even enhancer-specific.

### Evolution of double minutes

In in vitro systems of induced drug resistance, double minutes were shown to evolve with increasing drug concentrations, but also simply by longer passaging, which reflects in large heterogeneity between double minutes within a tumor cell line (Carroll et al. 1988; Von Hoff et al. 1988, 1990; Schoenlein et al. 1992; Coquelle et al. 1998; Singer et al. 2000; L’Abbate et al. 2014; Shoshani et al. 2021). That extrachromosomal structures can evolve was first suggested based on the observation that submicroscopic double minutes, also referred to as “episomes,” were detected in early passages of cell lines undergoing drug resistance induction (Von Hoff et al. 1988). Over time, these submicroscopic double minutes gradually enlarge and become visible by light microscopy (Carroll et al. 1988; Singer et al. 2000). Similarly, there is evidence that enlargement of double minutes may provide a selective advantage, as size was found to correlate with their copy number (Koche et al. 2020). In addition to increasing length, an increase in the number of gene copies was predicted to provide a selective advantage by in silico modeling, where double minutes with 2 copies of resistance gene were selected over double minutes with 1 copy (Shoshani et al. 2021). Indeed, a stepwise increase in selective pressure was paired with the evolution to double minutes with up to tenfold more gene copies per molecule (Shoshani et al. 2021). Thus, enlargement and increase in gene copy number provide double minutes with a selective advantage.

The evolution of double minutes is mediated by DNA breakage. Induction of fragile sites within double minutes has been reported to drive their evolution (Coquelle et al. 1998). To date, two mechanisms have been postulated to explain how DNA breakage could induce double minutes to evolve. First, their evolution could be caused by the selective incorporation of double minutes in micronuclei (see the “Elimination of double minutes” section), followed by DNA breakage and re-ligation (Shoshani et al. 2021). In this model, ligation of DNA fragments originating from various double minutes could generate complex novel sequences. A second possibility is that their evolution happens through multiple steps of inter-double minute fusions, possibly facilitated by their open chromatin landscape and/or the fact that double minutes end up in the same region of the nucleus during the S and G_2_ phases when HR is active (Kanda et al. 2001; Rothkamm et al. 2003; L’Abbate et al. 2014). Interestingly, a model describing the role of homologous recombination in double minute evolution has been put forward (Rosswog et al. 2021).

Selective integration of plasmid and viral DNA into pre-existing double minutes was repeatedly observed (Kanda et al. 2001; Shimizu et al. 2005, 2007). Recently, the existence of double minutes of combined human and viral origin was confirmed in HPV-positive oropharyngeal cancer (Pang et al. 2021). It seems that spatial proximity of double minutes and viral DNA as well as among double minutes is a pre-condition for their rearrangements. We postulate that they could get in such proximity in micronuclei or within restricted regions of the main nucleus. Taken together, reports show that double minutes appear to evolve via two distinct mechanisms that both depend on DNA breakage: (1) micronucleus disruption followed by DNA shattering, ligation, and subsequent reincorporation into the primary nucleus or (2) nuclear DNA breakage followed by inter-double minute fusions.

### The complex relationship of double minutes and HSRs

In juxtaposition to double minutes, the cytogenetic analyses of the last century defined homogeneously staining regions (HSRs). These amplifications are intrachromosomal and owe their name to the abnormal labeling pattern showing an absence of banding in karyotypic analyses (Levan et al. 1977). Both double minutes and HSRs have been described in in vitro systems of induced drug resistance (Haber and Schimke 1981; Singer et al. 2000), but also tumor-derived cancer cell lines (George and Powers 1982; Alitalo et al. 1983; Rosswog et al. 2021) and many tumor types (Turner et al. 2017). Interestingly, already in the early reports, it was proposed that these two ways of amplification could be related or interconvertible.

Both sides of the genomic amplification coin — intrachromosomal HSRs and extrachromosomal double minutes — can form through BFB cycles. HSRs were shown to form as a result of multiple iterations of BFB cycles (Cowell and Miller 1983; Coquelle et al. 1997; Shimizu et al. 2003, 2005). As mentioned previously and depicted in Fig. 3b, double minutes can form as a consequence of BFB cycles — either through direct ligation of broken DNA fragments or following micronucleation and subsequent re-incorporation of broken DNA fragments into the primary nucleus (Toledo et al. 1992; Singer et al. 2000; Rausch et al. 2012; Nones et al. 2014; Shoshani et al. 2021). Numerous studies described that HSRs can be converted to double minutes. Inducing DSBs in an HSR led to the generation of double minutes harboring the same gene (Coquelle et al. 2002). Further, treating HSR-containing cells with increasing concentrations of methotrexate (MTX) resulted in the loss of the HSR and acquisition of double minutes encoding *DHFR*, the target of MTX. Additionally, evidence to strongly support a model wherein BFB-mediated breakdown of HSRs leads to double minute formation has been gathered. HSRs were frequently observed to form DNA bridges (Shoshani et al. 2021; Singer et al. 2000). Moreover, live-cell imaging revealed that rupture of *DHFR* + anaphase bridges resulted in the production of *DHFR* + micronuclei, and whole-genome sequencing revealed that the double minutes were made up of several non-adjacent regions of the HSR (Shoshani et al. 2021). In conclusion, chromatid rupture as a consequence of repeated BFB cycles can result in the formation of double minutes, and evidence is emerging that this process could be specific to the BFB-mediated breakdown of HSRs.

Interestingly, the reverse process, HSRs being formed by double minute integration into the chromosomes, is also reported (discussed in the next section). Then, HSRs can act as (latent) reservoirs of double minutes, serving to generate new double minutes that can be positively selected for once selective pressures are in favor (Levan et al. 1977; Singer et al. 2000; Nathanson et al. 2014). As most integrations of double minutes into chromosomes occur near chromosome ends (Kaufman et al. 1983; Ruiz and Wahl 1990; Shoshani et al. 2021), this supports the idea that BFB-cycles are at the base of double minute re-generation.

A great number of past and recent studies addressed this complex relationship. When looked at collectively, it becomes clear that the fitness benefits these two types of amplification may confer are dictated by cell type and intricate environmental conditions the cells experience. Recent comprehensive analyses show double minutes are in some instances present concurrently with HSRs, while in other cases, the amplification is found to be exclusively extrachromosomal (Turner et al. 2017; Kim et al. 2020; Shoshani et al. 2021). This raises the question of the factors that affect this balance and differentially stabilize the two amplification types, how frequent these events of conversion are, and how they are regulated.

## Elimination of double minutes

Since double minutes are uniquely present in cancer cells, their elimination provides an attractive opportunity for therapeutic intervention. It was shown that double minutes or double minute-containing cells can be eliminated by various treatments (Table 2). Hence, exploiting the vulnerabilities of tumors with oncogenes amplified on double minutes is a strategy that merits further exploration.

**Table 2:**
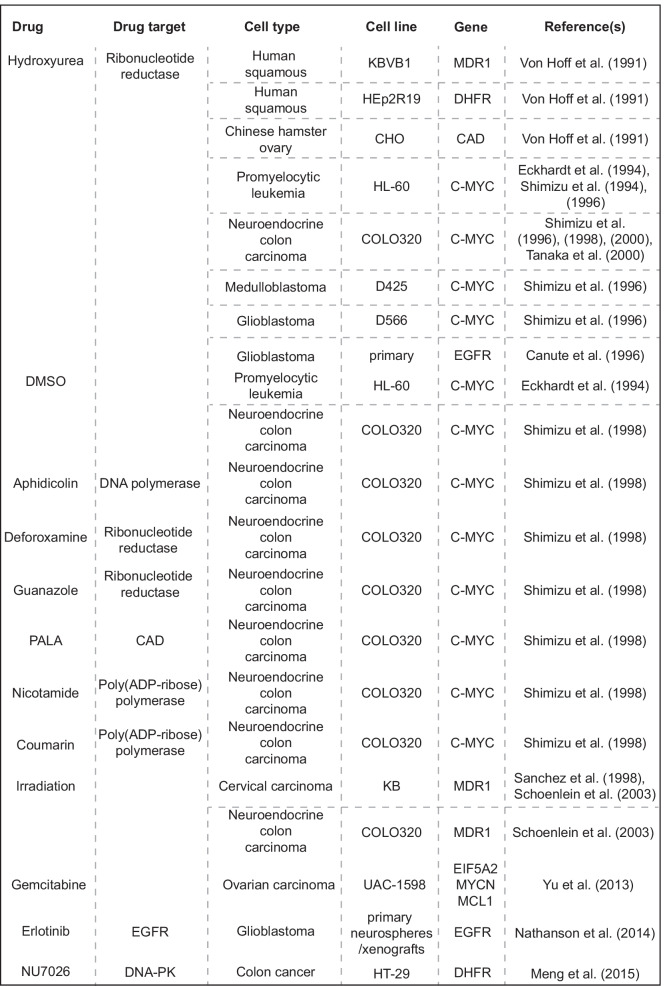
Overview of therapies that were shown to eliminate double minutes

### Elimination of double minutes through HSR formation

Double minutes can be depleted when the selective advantage that they confer to a cell is lost. This was frequently observed upon continued culturing in medium from which the selective agent has been withdrawn (Levan et al. 1977; Haber and Schimke 1981; Lin et al. 1985; Carroll et al. 1988; Ruiz and Wahl 1990; Schulte et al. 2012) and was also shown to occur in vivo in response to cancer therapy (Nathanson et al. 2014). A reversible model of drug resistance was established in glioblastoma patient-derived neurospheres. In this model, *EGFRvIII* amplified on double minutes was quickly lost in response to EGFR inhibitor treatment. Relatively quickly upon drug withdrawal, double minutes recurred (Nathanson et al. 2014).

**Fig. 6:**
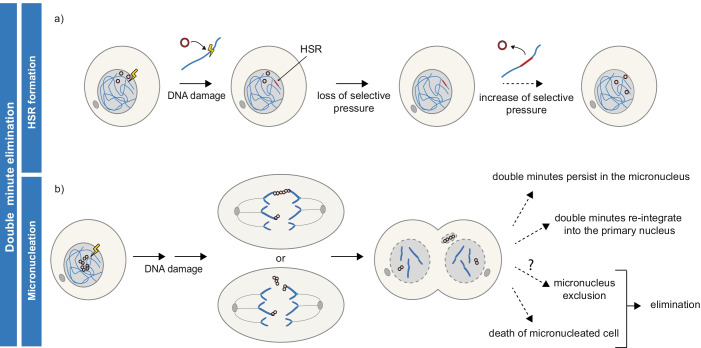
Routes of double minute elimination. **a** Double minutes are eliminated from cells when they integrate into a chromosome, thereby forming an HSR. Proposedly, double minute integration is a random event that is selected for when selective pressure is lost. Formed HSRs may serve as reservoirs of double minutes, generating new double minutes when selective pressures are in favour. **b** The fate of micronuclei containing double minutes are manifold. Double minutes may be lost through micronucleation, through cell death or other not fully characterized mechanisms

Reversible loss of double minutes can be explained by their integration into HSRs (Fig. 6a) (Lin et al. 1985; Carroll et al. 1988; Ruiz and Wahl 1990; Coquelle et al. 1998; Baiker et al. 2000; Shimizu 2009; Vogt et al. 2014; L’Abbate et al. 2014; Turner et al. 2017; Rosswog et al. 2021; Shoshani et al. 2021). Indeed, in the in vitro studies cited above, HSR formation was detected concomitant with the loss of double minutes (Lin et al. 1985; Carroll et al. 1988; Ruiz and Wahl 1990; Nathanson et al. 2014), and sequence analysis confirmed the ability of double minutes to integrate into chromosomes (Turner et al. 2017; Rosswog et al. 2021; Song et al. 2021). The integration of double minutes into chromosomes can be mediated by damage-induced DNA breakage (Shoshani et al. 2021). The integration event itself seems to be mediated by NHEJ or MMEJ, as sequence homologies are lacking, whereas microhomologies and short insertions were present at double minute-integration sites (L’Abbate et al. 2014; Vogt et al. 2014). The role of NHEJ in double minute to HSR conversion is substantiated by recent reports where treatment with DNA-PK inhibitor reduced the frequency of this event (Song et al. 2021). Although evidence is emerging to explain the mechanism of double minute integration, it is unclear what prompts this conversion of double minutes to HSR. Proposedly, double minute integration is a random event that becomes dominant when selective pressure is altered (Storlazzi et al. 2010).

The observation that double minutes are lost spontaneously in absence of selective pressure or upon neutralization of selective advantage (e.g., by therapy targeting a DM-encoded gene) suggests that their maintenance comes with a fitness cost (Levan et al. 1977; Haber and Schimke 1981; Lin et al. 1985; Carroll et al. 1988; Ruiz and Wahl 1990; Schulte et al. 2012; Nathanson et al. 2014; Lange et al. 2021). Therefore, it may be that as long as an intrachromosomal amplification suffices for cell survival, it will remain the predominant means of gene amplification in the population. Not only loss of selective pressure, but also stable selective pressure may result in the selection of HSR-containing cells (Song et al. 2021). However, in a changing environment and under stronger selective pressure, the extrachromosomal amplification would reappear in the cell population. This will be due to higher plasticity, higher gene copy number, and gene expression that extrachromosomal amplification can reach, compared to intrachromosomal (Turner et al. 2017; Wu et al. 2019; Song et al. 2021).

### Elimination of double minutes through micronucleation

Even though double minutes are acentric, their segregation in mitosis is surprisingly successful, and the great majority of these structures are found within the nucleus. As mentioned previously, this is enabled by their association with chromatid tips during anaphase. However, some studies observed that double minutes can form bridges in a subset of divisions that consist of “strings” of double minutes (Kanda et al. 1998; Shimizu et al. 2007). Other studies on the mitotic behavior of double minutes observed their incorporation into micronuclei in various cancer cell lines (Levan and Levan 1978; Tanaka and Shimizu 2000) and tumor biopsy samples (Valent et al. 2001). In addition, this entrapment of double minutes in micronuclei was shown to be promoted by various DNA-damaging agents (Von Hoff et al. 1991; Eckhardt et al. 1994; Canute et al. 1996; Shimizu et al. 1998, 2007; Sanchez et al. 1998; Tanaka and Shimizu 2000; Schoenlein et al. 2003; Yu et al. 2013). When treating double minute-containing cell lines with hydroxyurea, it was reported that repair on double minutes may be delayed as compared to breaks on the chromosomal DNA (Shimizu et al. 2007). Intriguingly, a few studies (Shimizu et al. 2007; Oobatake and Shimizu 2020) revealed that micronucleation of double minutes can happen, even when the damage is repaired. Therefore, the mechanisms by which DNA damage enhances micronucleation of double minutes remain uncharacterized.

Post-mitotic micronucleation of double minutes could happen through their aggregation combined with the loss of their association with chromatid tips or through an increase in the frequency of double minute “bridge” formation (Fig. 6b). To speculate, the formation of double minute aggregates in response to DNA damage could occur through DNA repair-dependent capture of double minutes, e.g., formation of HR intermediates. Further, mechanisms relying on DNA damage-induced post-replicative loading of cohesin(-like-acting) protein(s) (Litwin et al. 2018) could affect double minutes differently when compared to chromosomal DNA (Borrie et al. 2017). Aggregate formation and micronucleation were reported upon DSB induction (Oobatake and Shimizu 2020) and were increased when DNA-PKcs (NHEJ) (Meng et al. 2015) but also BRCA1 (HR) (Cai et al. 2019) function was perturbed, strengthening the role of DNA repair in this process.

The fate of micronuclei was described to be manifold. They can persist for several cell divisions, they can get reintegrated into the primary nucleus, but they can also be eliminated from the cell (depicted in Fig. 6b and reviewed in Hintzsche et al. 2017). In case the micronuclear content reintegrates into the primary nucleus, double minutes could be maintained but may have an altered size and sequence due to DNA breakage and re-ligation (see the “Evolution of double minutes” section). When it comes to elimination of micronuclei, several mechanisms have been proposed, such as their enzymatic degradation, elimination of micronucleated cells by cell death, but also their physical exclusion from the cell (Hintzsche et al. 2017). The mechanism of physical exclusion was described specifically in the context of double minutes. Here, intact double minute-enriched micronuclei, proposed to be eliminated by cellular membrane blebbing, were detected in the culture fluid of double minute-containing cancer cells, showing that exclusion could indeed be a mode of their elimination (Shimizu et al. 2000; Oobatake and Shimizu 2020). These models are yet to be further examined*.*

Elimination of double minutes caused by DNA damage-induced micronucleation was shown to affect tumor cell properties (Von Hoff et al. 1991, 1992; Eckhardt et al. 1994; Shimizu et al. 1994, 1998, 2007; Canute et al. 1996; Sanchez et al. 1998; Schoenlein et al. 2003; Yu et al. 2013). For instance, hydroxyurea-induced depletion of double minutes led to cellular differentiation (Eckhardt et al. 1994) and severely reduced tumor-forming capacity in nude mice (Von Hoff et al. 1992). Besides hydroxyurea, other means of inducing DNA damage were also shown to be effective against double minutes. Treating ovarian cancer cells with gemcitabine resulted in micronuclei formation and a significant decrease in double minutes. As a consequence, tumorigenic potential, as measured in colony formation and invasion assays, decreased (Yu et al. 2013). Furthermore, induction of DNA damage by exposing cells to ionizing radiation resulted in micronuclear capture of double minutes and concomitant reduction in drug resistance (Sanchez et al. 1998; Schoenlein et al. 2003). Apart from these in vitro results, a study aiming to eliminate double minutes in patients with ovarian carcinoma by treatment with a non-cytotoxic dose of hydroxyurea was performed (Raymond et al. 2001). Hydroxyurea treatment resulted in a decrease in double minutes paired with increased progression-free survival in a proportion of subjects, demonstrating that double minute elimination may be beneficial for some groups of cancer patients. However, randomized, placebo-controlled studies are required to draw definitive conclusions about the efficacy of DNA damage induction as an anti-double minute therapy in vivo.

## Concluding remarks and future perspectives

Although double minutes/ecDNAs were discovered more than 50 years ago, their widespread implications for cancer biology were only recently acknowledged, igniting a burst of novel reports on the causes and consequences of their formation. Together with pioneering work dating back to the decades succeeding their initial discovery, these reports have provided important insights into the generation, maintenance, and elimination of double minutes. The life of double minutes begins with their formation, which can be relatively “simple” or be paired with gross chromosomal rearrangements. Double minutes are then maintained through replication that resembles the one of chromosomal DNA, followed by random distribution over daughter cells upon cell division. During their life, double minutes can evolve through entrapment to micronuclei, subsequent DNA breakage, and re-ligation and/or through fusing with other double minutes. Alternatively, micronucleation of double minutes can result in their irreversible elimination, possibly through micronucleus exclusion or cell death of the micronucleated cell. A second, reversible mechanism of double minute elimination is provided by their integration into the chromosomal DNA, in the form of an HSR, only to appear again when selective pressure is enhanced. Strikingly, many key processes of double minute biology are governed by DNA damage. Although DNA damage being at the base of their generation may be of no surprise, DNA damage also plays a role in their evolution, integration into chromosomes, and in their elimination through micronuclear capture. DNA damage regulating multiple steps of the lifecycle of double minutes brings along challenges, most importantly as in how to address double minutes in the clinic. For instance, double minutes were frequently described to arise in response to drug treatments in in vitro systems (Hahn et al. 1987; Toledo et al. 1993; Coquelle et al. 1997), but also in patients (Shoshani et al. 2021), thereby conferring resistance to therapy. However, they were also shown to be depleted in response to therapy targeting the gene encoded on them, thereby showing that the presence of double minutes in a tumor can be exploited in treatment (Nathanson et al. 2014). Even a single drug, hydroxyurea, can both be used to stimulate the generation of double minutes (Mariani and Schimke 1984; Hill and Schimke 1985) and to decrease their levels (Von Hoff et al. 1992; Shimizu et al. 1998). Thus, a tight balance between generation, maintenance, and elimination of double minutes exists.

Many aspects of double minute/ecDNA biology await further investigation. What makes a tumor susceptible to double minute generation and maintenance, or in other words, how do tumor properties such as cell type, mutational landscape, and possibly chromosomal instability status correlate with the presence of double minutes? Are there regions in the genome that are more prone to engage in amplification or are specific amplifications simply products of selective pressure? What mechanism(s) underlie double minute generation without concomitant chromosomal rearrangements? Which cellular proteins do double minutes exploit to ensure their maintenance and expression, and how? What underlies the differences between chromosomal DNA and double minutes concerning DNA damage repair? How do double minutes form clusters, and how does cluster formation affect their maintenance and function? And what about the tumor-microenvironment? Are double minute-containing cells recognized by the immune system, for example, through the generation of neo-antigens or activation of signaling cascades such as the cGAS-STING pathway?

Ultimately, gaining insight into matters such as these will improve our understanding of double minutes/ecDNA as a unique feature of cancer, which may lead to the development of widely applicable therapies that specifically combat tumor tissue, while leaving the healthy tissue unaffected.

## Supplementary Information

Below is the link to the electronic supplementary material.

Supplementary file1 (PDF 2216 KB)

## Data Availability

Not applicable.
