# Supplementary material for: Life of double minutes: generation, maintenance, and elimination
Source: Chromosoma. 2022 Apr 30;131(3):107–25. doi: 10.1007/s00412-022-00773-4 (PMC9470669; doi:10.1007/s00412-022-00773-4)
Supplement: Supplementary file 1 — Supplementary file1 (PDF 2216 KB) [file 412_2022_773_MOESM1_ESM.pdf]

Adis Bohn Stafleu van Loghum palgrave macmillan Springer

Change of authorship request form (pre-acceptance)

Please read the important information on page 4 before you begin

This form should be used by authors to request any change in authorship including changes in corresponding authors. Please fully complete all sections. Use black ink and block capitals and provide each author's full name with the given name first followed by the family name.

Please note: In author collaborations where there is formal agreement for representing the collaboration, it is sufficient for the representative or legal guarantor (usually the corresponding author) to complete and sign the Authorship Change Form on behalf of all authors.

**Manuscript ID no. in case of unpublished manuscript:** CHSO-D-21-00035R1	
**DOI in case of published manuscript:**	
**ISBN (for books):**	

Section 1: Please provide the current title of manuscript
(For journals: Please provide the manuscript ID, title and/or DOI if available.)
(For books: Please provide the title, ISBN and/or DOI if available.)

Title: Life of double minutes: Generation, maintenance and Elimination

Section 2: Please provide the previous authorship, in the order shown on the manuscript before the changes were introduced. Please indicate the corresponding author by adding (CA) behind the name.

	First name(s)	Family name	ORCID or SCOPUS id, if available
1st author	IRENE C.	ZAALBERG	0000-0001-5535-2853
2nd author	MILA	ILIĆ	0000-0003-4664-9393
3rd author	JONNE A.	RAAIJMAKERS	0000-0003-1759-8347
4th author	RENÉ H.	MEDEMA (CA)	0000-0002-6754-0381
5th author			
6th author			
7th author			

Please use an additional sheet if there are more than 7 authors.

SPRINGER NATURE Springer Nature is one of the world's leading global research, educational and professional publishers, created in May 2015 through the combination of Nature Publishing Group, Palgrave Macmillan, Macmillan Education and Springer Science+Business Media.

Change of authorship request form (pre-acceptance)

Section 3: Please provide a justification for change. Please use this section to explain your reasons for changing the authorship of your manuscript, e.g. what necessitated the change in authorship? Please refer to the (journal) policy pages for more information about authorship. Please explain why omitted authors were not originally included and/or why authors were removed on the submitted manuscript.

In the first place, I would like to clarify that no authors have been added or removed from the author's list. Like in the first submission, this manuscript has two co-authors sharing the first authorship. In the final submission, the order of these co-authors has changed, since one of them took a dominant role when it came to addressing the reviewer's comments. Having this in mind and since Mila Ilić was the one that conceptualized the manuscript, I found this change appropriate. This change has been communicated to and accepted by all the authors.

Section 4: Proposed new authorship. Please provide your new authorship list in the order you would like it to appear on the manuscript. Please indicate the corresponding author by adding (CA) behind the name. <u>If the corresponding author has changed, please indicate the reason under section 3.</u>

	First name(s)	Family name (this name will appear in full on the final publication and will be searchable in various abstract and indexing databases)
1st author	MILA	ILIĆ
2nd author	IRENE C.	ZAALBERG
3rd author	JONNE A.	RAAIJMAKERS
4th author	RENÉ H.	MEDEMA (CA)
5th author		
6th author		
7th author		

Please use an additional sheet if there are more than 7 authors.

SPRINGER NATURE Springer Nature is one of the world's leading global research, educational and professional publishers, created in May 2015 through the combination of Nature Publishing Group, Palgrave Macmillan, Macmillan Education and Springer Science+Business Media.

Change of authorship request form (pre-acceptance)

Section 5: Author contribution, Acknowledgement and Disclosures. Please use this section to provide a new disclosure statement and, if appropriate, acknowledge any contributors who have been removed as authors and ensure you state what contribution any new authors made (if applicable per the journal or book (series) policy). Please ensure these are updated in your manuscript - after approval of the change(s) - as our production department will not transfer the information in this form to your manuscript.

New acknowledgements:

This work was supported by Oncode.
We thank Piet Borst for the critical reading of the manuscript.

New Disclosures (financial and non-financial interests, funding):

The authors declare no conflict of interest.
This work was supported by Oncode (project number 59043).

New Author Contributions statement (if applicable per the journal policy):

M.I. and I.C.Z. interpreted the studies and wrote the manuscript. M.I. and I.C.Z. contributed equally and reserve the right to list themselves first on the author's list. J.A.R. and R.H.M. reviewed and made significant changes to the manuscript.

State 'Not applicable' if there are no new authors.

SPRINGER NATURE Springer Nature is one of the world's leading global research, educational and professional publishers, created in May 2015 through the combination of Nature Publishing Group, Palgrave Macmillan, Macmillan Education and Springer Science+Business Media.

Adis | Bohn Stafleu van Loghum | palgrave macmillan | Springer

Change of authorship request form (pre-acceptance)

Section 6: Declaration of agreement. *All* authors, unchanged, new and removed *must* sign this declaration.
(NB: Please print the form, sign and return a scanned copy. Please note that signatures that have been inserted as an image file are acceptable as long as it is handwritten. Typed names in the signature box are unacceptable.) *** Please delete as appropriate. Delete all of the bold if you were on the original authorship list and are remaining as an author.**

	First name	Family name		Signature	Affiliated institute	Date
1st author	Mila	Ilić	I agree to the proposed new authorship shown in section 4 **/and the addition/removal*of my name to the authorship list.**			
2nd author	Irene C.	Zaalberg	I agree to the proposed new authorship shown in section 4 **/and the addition/removal*of my name to the authorship list.**			
3rd author	Jonne A.	Raaijmakers	I agree to the proposed new authorship shown in section 4 **/and the addition/removal*of my name to the authorship list.**			
4th authors	René H.	Medema	I agree to the proposed new authorship shown in section 4 ~~**/and the addition/removal*of my name to the authorship list.**~~	[signature]	Netherlands Cancer Institute	17.3.2022.
5th author			I agree to the proposed new authorship shown in section 4 **/and the addition/removal*of my name to the authorship list.**			
6th author			I agree to the proposed new authorship shown in section 4 **/and the addition/removal*of my name to the authorship list.**			
7th author			I agree to the proposed new authorship shown in section 4 **/and the addition/removal*of my name to the authorship list.**			

Please use an additional sheet if there are more than 7 authors.

SPRINGER NATURE Springer Nature is one of the world's leading global research, educational and professional publishers, created in May 2015 through the combination of Nature Publishing Group, Palgrave Macmillan, Macmillan Education and Springer Science+Business Media.

Adis Bohn Stafleu van Loghum palgrave macmillan Springer

Change of authorship request form (pre-acceptance)

Important information. Please read.

- Please return this form, fully completed, to Springer Nature. We will consider the information you have provided to decide whether to approve the proposed change in authorship. We may choose to contact your institution for more information or undertake a further investigation, if appropriate, before making a final decision.
- By signing this declaration, all authors guarantee that the order of the authors are in accordance with their scientific contribution, if applicable as different conventions apply per discipline, and that only authors have been added who made a meaningful contribution to the work.
- Please note, we cannot investigate or mediate any authorship disputes. If you are unable to obtained agreement from all authors (including those who you wish to be removed) you must refer the matter to your institution(s) for investigation. Please inform us if you need to do this.
- If you are not able to return a fully completed form within **30 days** of the date that it was sent to the author requesting the change, we may have to withdraw your manuscript. We cannot publish manuscripts where authorship has not been agreed by all authors (including those who have been removed).
- Incomplete forms will be rejected.

SPRINGER NATURE Springer Nature is one of the world's leading global research, educational and professional publishers, created in May 2015 through the combination of Nature Publishing Group, Palgrave Macmillan, Macmillan Education and Springer Science+Business Media.
